# Foliar application of strigolactones improves the desiccation tolerance, grain yield and water use efficiency in dryland wheat through modulation of non-hydraulic root signals and antioxidant defense

**DOI:** 10.1007/s44154-023-00127-9

**Published:** 2023-12-06

**Authors:** Sha Guo, Xiaofei Wei, Baoluo Ma, Yongqing Ma, Zihan Yu, Pufang Li

**Affiliations:** 1 College of Forestry, Northwest A&F University, Shaanxi, 712000 Yangling China; 2 College of Soil and Water Conservation Science and Engineering, Northwest A&F University, Shaanxi, 712000 Yangling China; 3 Ottawa Research and Development Centre (ORDC), Agriculture and Agri-Food Canada, Ottawa, ON K1A0C6 Canada; 4 College of Natural Resources and Environment, Northwest A&F University, Shaanxi, 712000 Yangling China

**Keywords:** Strigolactones, Non-hydraulic root signals, Antioxidant defense system, Grain yield, Water use efficiency

## Abstract

Non-hydraulic root signals (nHRS) are affirmed as a unique positive response to soil drying, and play a crucial role in regulating water use efficiency and yield formation in dryland wheat production. Strigolactones (SLs) can enhance plant drought adaptability. However, the question of whether strigolactones enhance grain yield and water use efficiency by regulating nHRS and antioxidant defense systems in dryland wheat remains unanswered. In this study, pot experiments were conducted to investigate the effects of strigolactones on nHRS, antioxidant defense system, and grain yield and water use efficiency in dryland wheat. The results showed that external application of SLs increased drought-induced abscisic acid (ABA) accumulation and activated an earlier trigger of nHRS at 73.4% field capacity (FC), compared to 68.5% FC in the control group (CK). This phenomenon was mechanically associated with the physiological mediation of SLs. The application of SLs significantly enhanced the activities of leaf antioxidant enzymes, reduced ROS production, and mitigated oxidative damage to lipid membrane. Additionally, root biomass, root length density, and root to shoot ratio were increased under strigolactone treatment. Furthermore, exogenous application of SLs significantly increased grain yield by 34.9% under moderate drought stress. Water use efficiency was also increased by 21.5% and 33.3% under moderate and severe drought conditions respectively, compared to the control group (CK). The results suggested that the application of strigolactones triggered earlier drought-sensing mechanism and improved the antioxidant defense ability, thus enhancing grain yield and water use efficiency in dryland wheat production.

## Introduction

Wheat is a staple food for more than 35% of the world population and is also the second grain crop in China. Its production status is directly related to social stability, the national economy, the people's livelihood and sustainable development in northwestern China (Xiao et al. 2022). Currently, drought is an important factor limiting crop production in arid and semi-arid environments, and 45% of the wheat area sown in developing countries is periodically affected by drought (Ge et al. 2016; Batool et al. 2019). Drought stress can reduce wheat yields by up to 50% (Reynolds et al. 2007; Fang et al. 2017) due to significant reductions in plant growth, photosynthetic rate, shoot biomass, and increase in oxidative damage (Ehdaie et al. 2008, 2012). Plants have developed various kinds of biochemical and physiological mechanisms to cope with temporary or terminal water shortage (Turner et al. 2014; Seleiman et al. 2021). There are pre-existing and induced defense mechanisms that adapt to drought stress conditions (Du et al. 2012; Lv et al. 2019).

In drying soil, an early response of plant is the closure of stomata without a detectable change in wheat leaf water status through the regulation of drought-induced abscisic acid (ABA) (Zhang et al. 2022). This response is perceived from the roots and then transmitted with chemical signals to the shoot (Jakab et al. 2005; Du et al. 2012). The early-warning of wheat plant response to soil drying is defined as a non-hydraulic root signal (nHRS) according to the root-shoot communication theory (Du et al. 2012; Lv et al. 2019). The early response of nHRS will help the wheat plant reduce stomatal water loss and is considered the first line of defense against possible drought stress (Lv et al. 2019). With the prolongation of soil water deficit, leaf stomatal conductance (*gs*) further decreases and leaf water content decreases significantly, a condition defined as a hydraulic root signal (HRS). The appearance of the hydraulic root signal is accompanied by significant changes in the osmotic adjustment and antioxidant defense mechanisms (Du et al. 2012; Lv et al. 2019). Wheat plants reduce the osmotic potential through osmoregulation to enhance their ability to absorb and retain water (Li et al. b). Soluble sugars and proline have been considered to cause osmotic adjustment under drought stress conditions (Ruszkowski et al. 2015; Li et al. b). Drought stress leads to reduced CO_2_ assimilation, decreased electron transmission, and the accumulation of reactive oxygen species (ROS). ROS are highly reactive chemical molecules that induced oxidative stress on proteins, membrane lipids and other cellular components in wheat (Wang et al. 2019). Plants have developed enzymatic antioxidant systems to overcome oxidative damage. These enzymes, including superoxide dismutase (SOD), ascorbate peroxidase (APX), catalase (CAT) and glutathione reductase (GR), can scavenge and prevent the overproduction of ROS. There exists a threshold range of soil moisture from the commencement to the end of nHRS, in which nHRS plays a series of critical regulatory roles (Xiong et al. 2006a; Lv et al. 2019). The threshold range of nHRS is closely related to wheat yield and water use efficiency (Lv et al. 2019). Wheat cultivars with wider threshold range of nHRS can induce earlier closure of leaf stomata, reduce leaf lethal water potential and reactive oxygen, and optimize root structure, resulting in higher water use efficiency and grain yield (Xiong et al. 2006a; Du et al. 2012; Lv et al. 2019).

Strigolactones (SLs) are a new class of phytohormones that are sensitive to drought stress and participate in the rapid response of related signaling pathways (Zhao et al. 2021). They are among the newly discovered important endogenous mediators in recent years to regulate drought adaptation in crops (Min et al. 2019; Raza et al. 2022). Many studies have found that SLs participate in regulating the physiological and ecological response to drought stress, and play an important role in promoting the drought adaptability of plants (Li et al. 2019; Xu et al. 2023). For example, SLs have been reported to improve the sensitivity of plant leaves to ABA and induce the synthesis and secretion of ABA, thereby regulating stomatal closure and improving the drought resistance of crops (Min et al. 2019). SLs also can inhibit the accumulation of ROS and malondialdehyde (MDA) and increase the antioxidant enzyme activity (Xu et al. 2023; Yang et al. 2023). Although the effect of SLs on improving plant drought resistance has been widely reported, whether SLs can increase the threshold range of nHRS remains to be confirmed. It is not clear whether SLs increase water use efficiency, dehydration tolerance, and grain yield by initiating the antioxidant defense mechanisms. Therefore, we conducted two pot studies in order to (1) evaluate the effect of SLs on the threshold range of nHRS and antioxidant defense mechanism; and (2) identify the effects of SLs on the desiccation tolerance, water use efficiency and grain yield of dryland wheat. We hypothesized that SLs can trigger ABA accumulation and earlier drought-sensing, and improve antioxidant defense ability, thereby increasing grain yield and water use efficiency under drought stress.

## Results

### Effects of SLs on ABA concentration

**Fig. 1:**
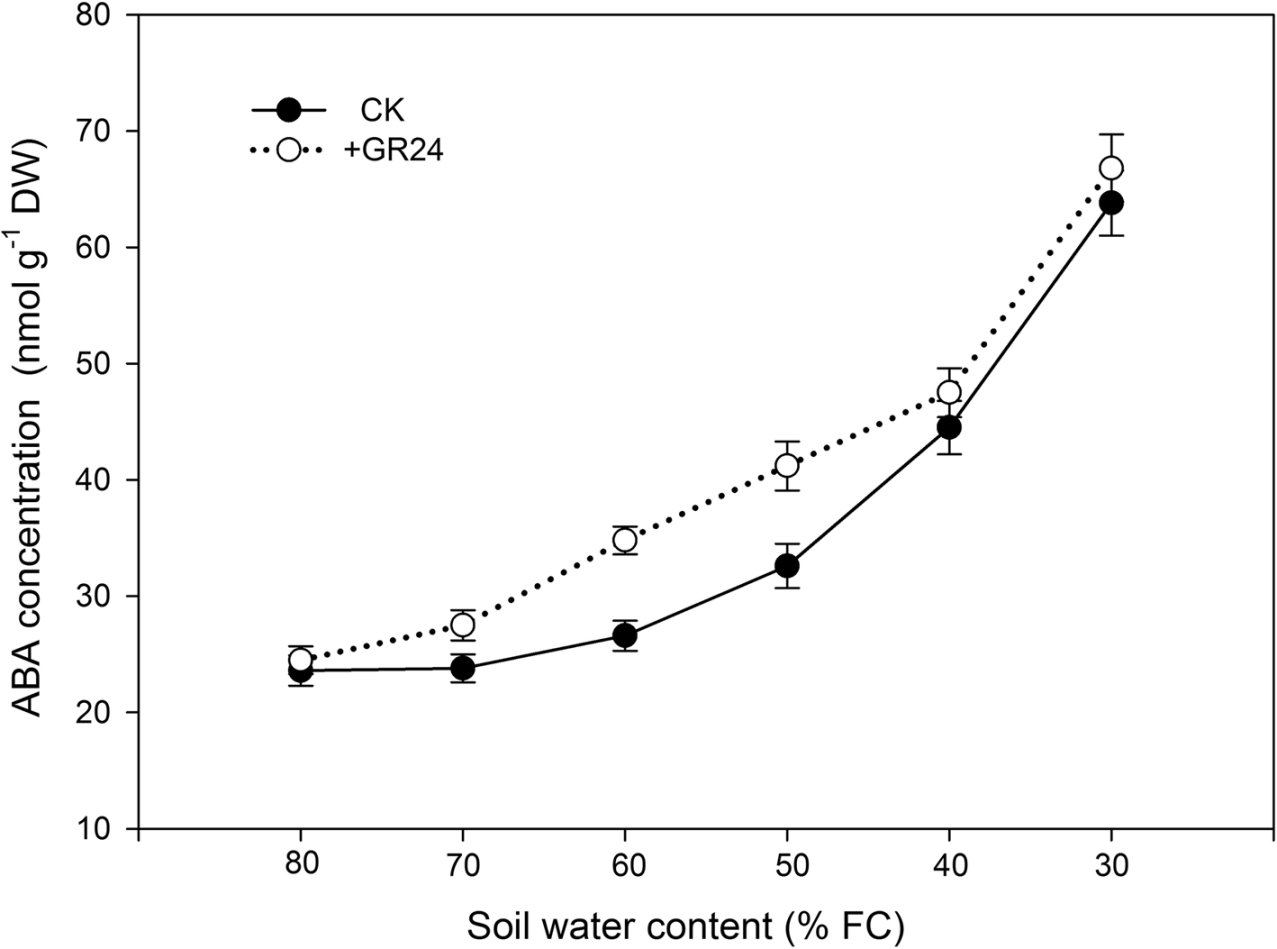
Impact of strigolactone application on abscisic acid (ABA) concentration in the leaves of dryland wheat under progressive soil drying conditions

GR24, synthetic analogues of SLs, was applied as a foliar spray to plants under different soil water content (SWC). Subsequently, leaves were collected and analyzed for abscisic acid (ABA) concentration. The ABA concentration in leaves of dryland wheat sprayed with and without GR24 has a significant difference when the soil water content was at 70% field capacity (FC) (Fig. 1). When the soil water content fell to 60% and 50% FC, the ABA concentration in leaves treated with GR24 was notably higher than in untreated leaves. Specifically, the ABA concentration in GR24-treated leaves was 34.6 nmol g^−1^ DW and 41.6 nmol g^−1^ DW, marking an increase of 30.6% and 28.9% respectively compared to the control group at soil water content levels of 60% and 50% FC. However, as the soil water content further decreased to 40% and 30% FC, the ABA concentration in the leaves showed no significant difference between the GR24-treated and untreated groups (Fig. 1).

### Effects of SLs on *gs* and RWC

**Fig. 2:**
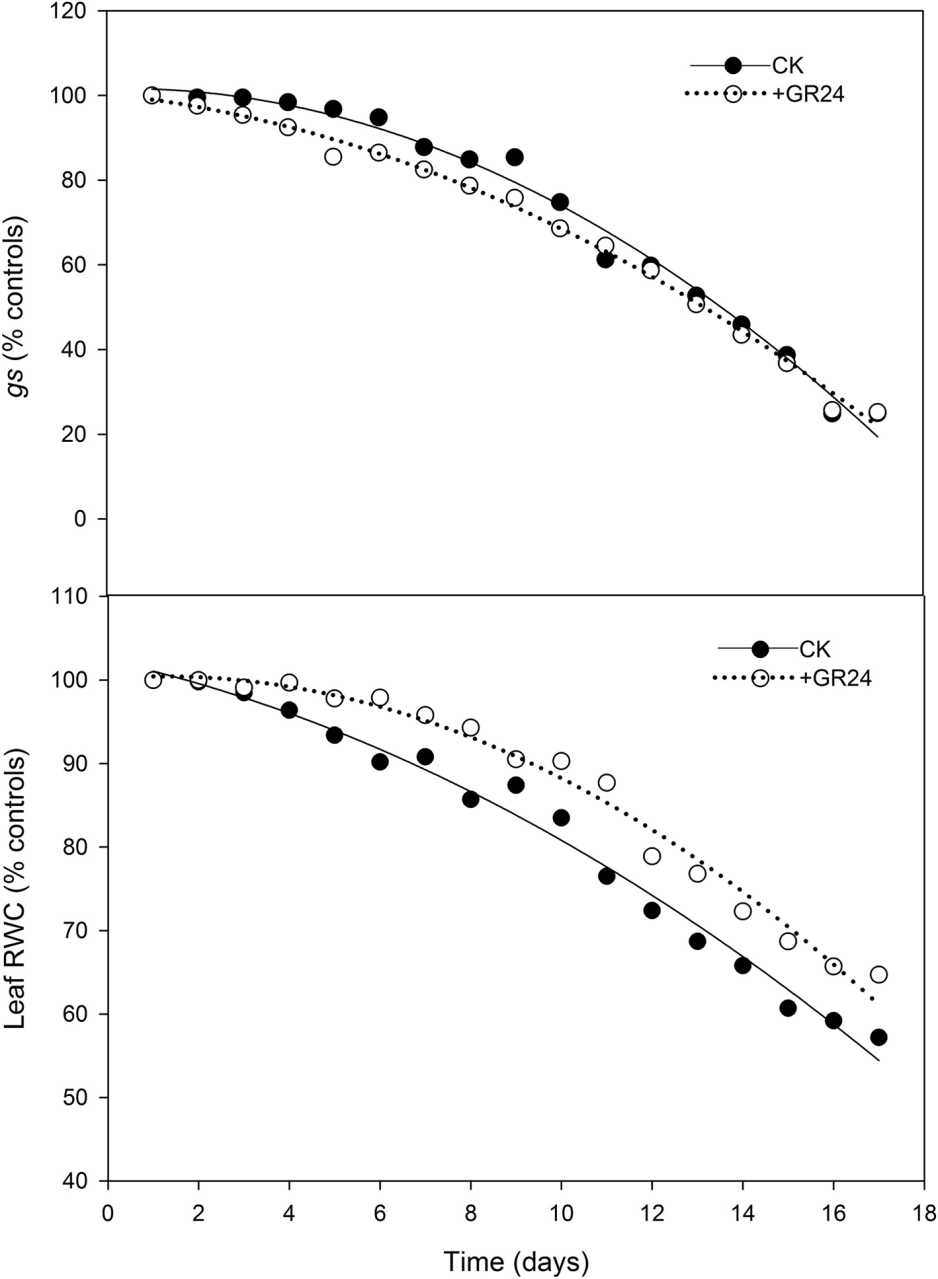
Impact of strigolactone treatment on stomatal conductance (*gs*) and leaf relative water content (RWC) in dryland wheat under water stress conditions. After the cessation of watering, the changes in stomatal conductance (*gs*) and leaf relative water content (RWC) in dryland wheat with different treatments were measured and expressed as a percentage of the control (CK)

**Fig. 3:**
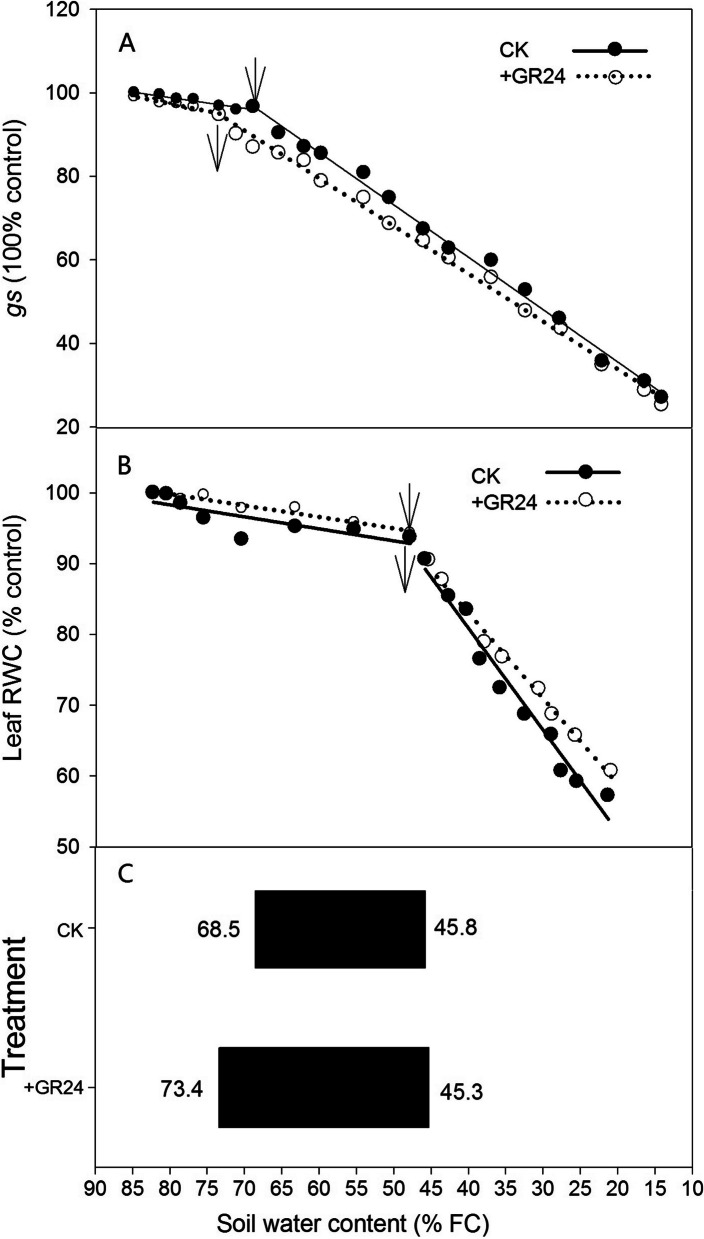
Strigolactone treatment alters soil water content (SWC) thresholds for stomatal conductance (*gs*) and leaf water content (RWC) in dryland wheat. Correlationship between *gs* (% CK) and SWC (**A**) and correlationship between leaf RWC (% CK) and SWC (**B**) were fitted by a linear-plateau function. Arrows indicate the decreasing points of *gs* and RWC in dryland wheat. (**C**) The SWC thresholds between the *gs*-decreasing-point and the leaf RWC-decreasing-point, with or without strigolactone treatment, in dryland wheat

The stomatal conductance (*gs*) and relative water content (RWC) in plants affected by drought, both with and without GR24 treatment, were charted over time. As shown in Fig. 2, results indicated a significant decrease in both leaf *gs* and RWC. In plants experiencing prolonged drought, *gs* typically decreased as soil water content progressively lowers, usually without a significant change in leaf RWC, suggesting an operation of non-hydraulic root signals (nHRS). The *gs* of leaves treated with GR24 declined earlier compared to those without GR24 treatment. In contrast, without GR24 treatment, leaf RWC decreased earlier than in the GR24-treated group. SWC thresholds for leaf *gs* and RWC, relative to the well-watered control, were determined by linear plateau functions (Fig. 3). When SWC was below the threshold, both *gs* and RWC exhibited a linear decrease. SWC thresholds for *gs* were 73.4% and 68.5% FC for GR24-treated and untreated groups, respectively, while the SWC thresholds for leaf RWC were 45.3% and 45.8%, respectively. Compared with the untreated group, the application of GR24 extended the SWC threshold range of dryland wheat from 68.5%–45.8% FC to 73.4%–45.3% FC, demonstrating the potential of GR24 in enhancing drought resilience.

**Fig. 4:**
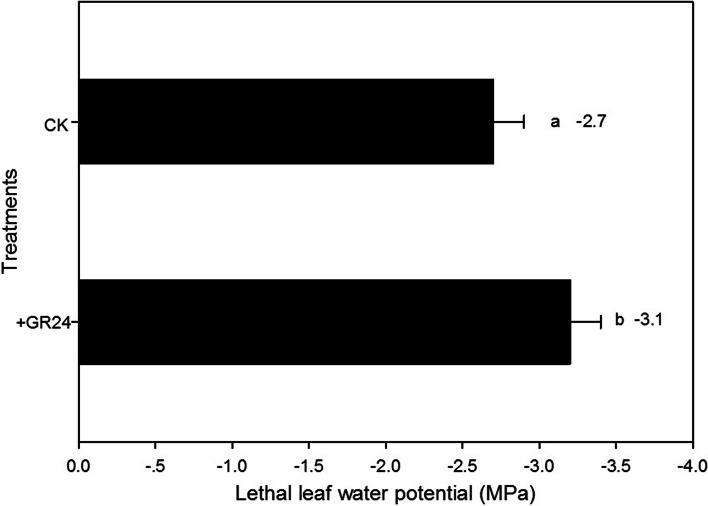
The effect of strigolactones on the lethal leaf water potential in dryland wheat

The application of GR24 significantly reduced lethal leaf water potential to -3.1 MPa, in comparison to -2.7 MPa in plants without GR24 treatment. This 14.8% decrease in the lethal water potential indicated that GR24 treatment enhanced the plant's tolerance to desiccation under soil drying conditions (Fig. 4).

### Reactive oxygen species (ROS) and lipid membrane peroxide levels

**Fig. 5:**
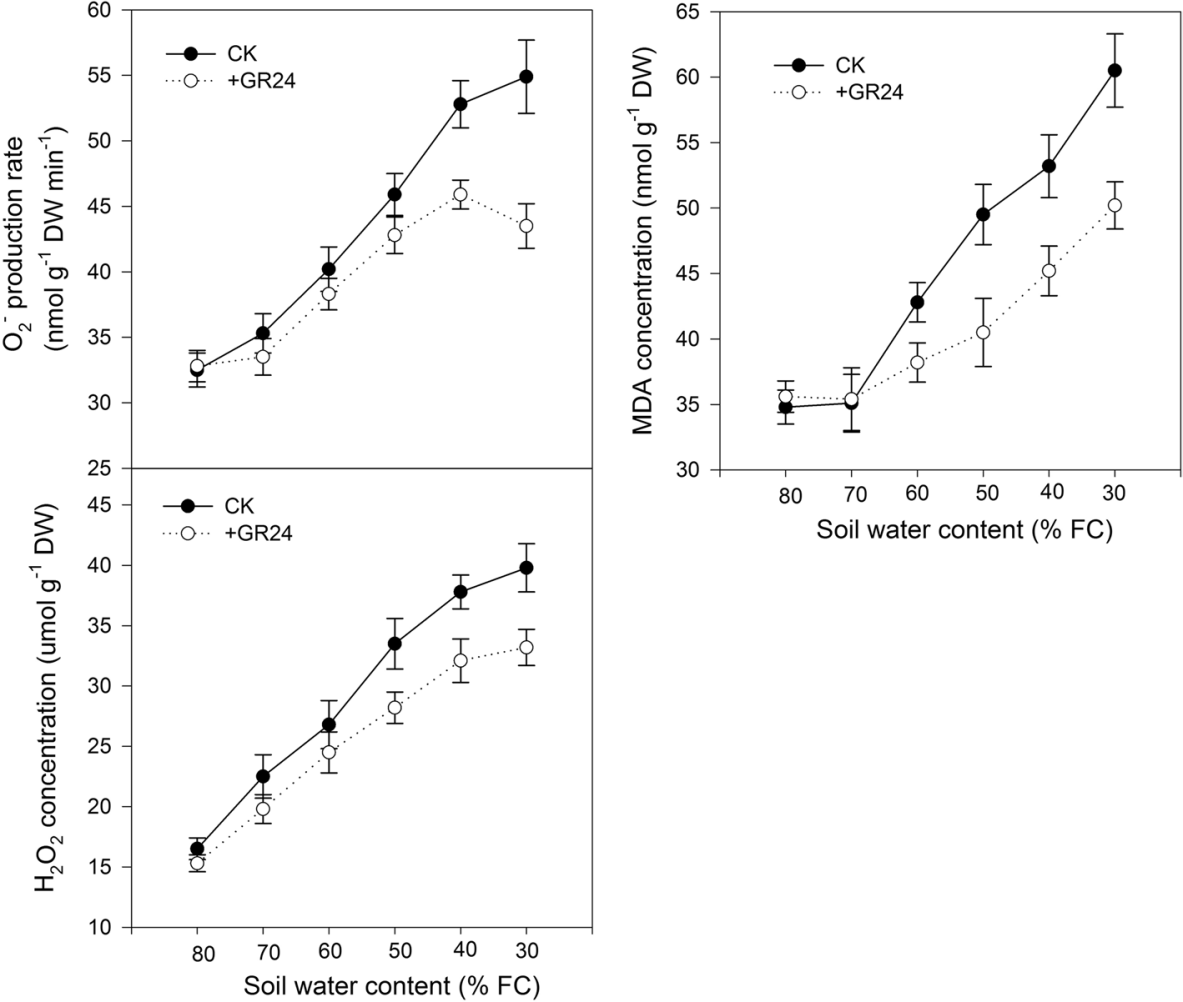
The effect of strigolactones on production rate of reactive oxygen species (O_2_^−^), hydrogen peroxide concentration (H_2_O_2_), and malondialdehyde (MDA) concentration in leaves of dryland wheat

In dryland wheat leaves, O_2_^−^ production rate and H_2_O_2_ accumulation increased progressively with soil drying (Fig. 5). As the SWC decreased below 50% FC, O_2_^−^ production rate in the leaves was significantly reduced by GR24 treatment. Specifically, when the SWC was dried to 30% FC, the O_2_^−^ production rate in the GR24-treated plants decreased significantly by 20.6% compared with CK. The effect of GR24 treatment on H_2_O_2_ accumulation was slightly different from its impact on O_2_^−^ production. When the SWC fell below 60% FC, H_2_O_2_ accumulation in the leave was significantly reduced by GR24 treatment. Lipid membrane peroxide, as indicated by Malondialdehyde (MDA) content, increased significantly with intensifying drought stress. However, GR24 treatment significantly reduced MDA content when SWC decreased to 60% FC, thereby mitigating the oxidative damage to membranes.

### Antioxidant enzyme activities

**Fig. 6:**
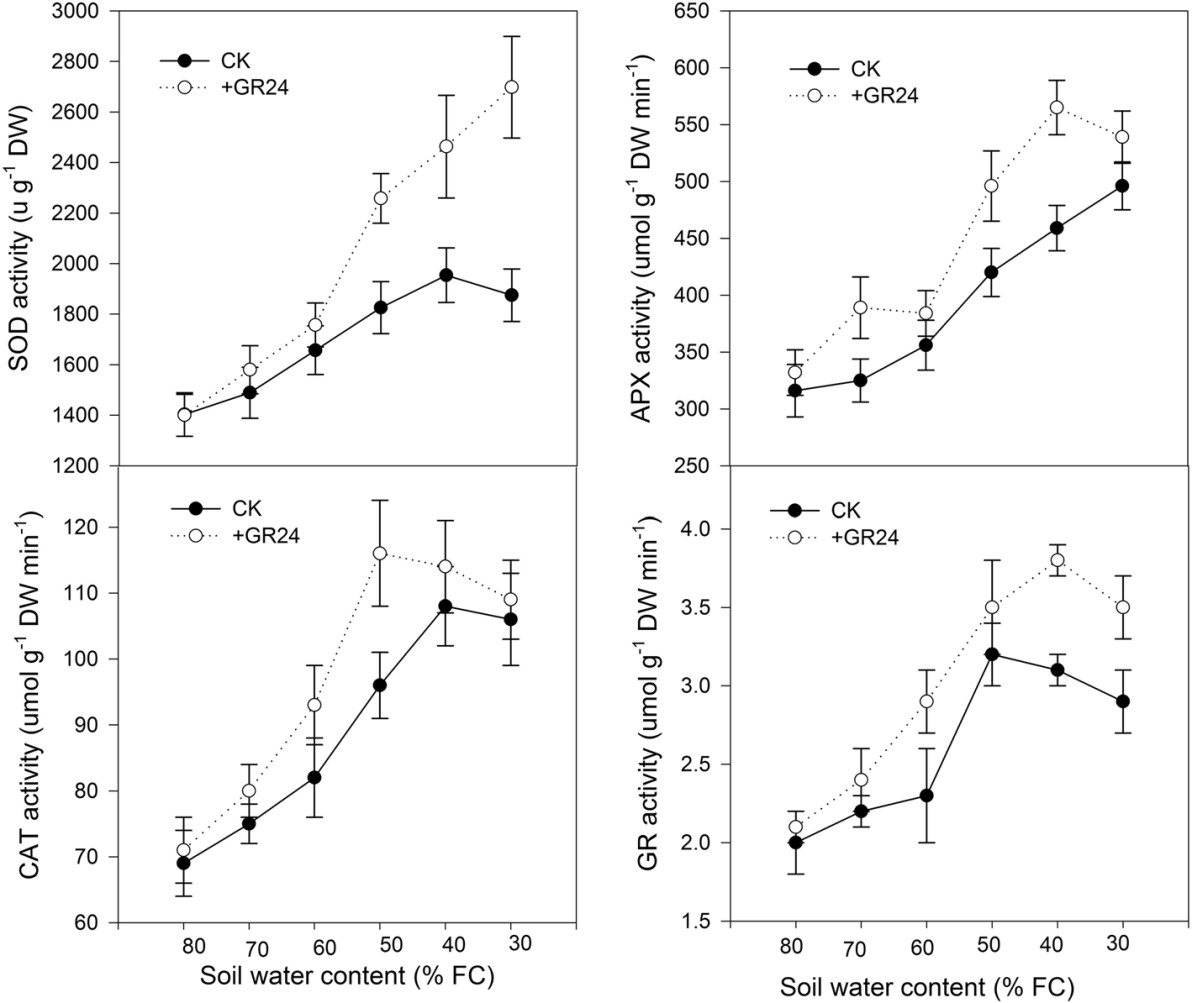
The effect of strigolactones on the activity of the antioxidant enzymes: superoxide dismutase (SOD), catalase (CAT), ascorbate peroxidase (APX), and glutathione reductase (GR) in leaves of dryland wheat

As drought stress intensifies, antioxidant enzyme activities rise. When soil water content (SWC) is above approximately 50% field capacity (FC), superoxide dismutase (SOD) activity shows no significant difference between treatments with and without GR24. However, when SWC decreases to 50% FC, SOD activity significantly increases under GR24 treatment compared to plants without GR24 treatment (Fig. 6). Similarly, ascorbate peroxidase (APX) activity is significantly higher in GR24-treated plants than in those without GR24 treatment until SWC drops below 50% FC. When SWC continues to decrease to severe water deficits (around 30% FC), no significant difference in APX activity is observed between GR24-treated and untreated plants. Catalase (CAT) activity is significantly higher in GR24-treated plants than in those without GR24 treatment, but only when SWC is at 50% FC. In contrast, glutathione reductase (GR) activity is significantly higher in GR24-treated plants than in those without GR24 treatment until SWC decreases to 40% FC (Fig. 6).

### Root morphological characters

Severe drought stress (30% FC) significantly decreased root biomass, total root length (TRL), root length density (RLD) and root surface area (RSA) without GR24 treatment (Table 1). In contrast, moderate drought stress (50% FC) significantly increased these root parameters, as well as the root to shoot ratio (RSR), in dryland wheat without GR24 treatment. Specifically, root biomass, total root length, root length density, root surface area and root to shoot ratio were 26.7%, 14.5%, 42.1%, 5.0% and 36.4% higher, respectively, under the moderate drought stress compared to well-watered condition. In general, GR24 treated wheat significantly increased root biomass, total root length, root length density, root surface area and root shoot ratio (RSR) in dryland wheat under both moderate and severe drought stress. The root biomass was 36.8% and 54.5% higher with GR24 treatment under moderate and severe drought stress, respectively, compared to plants with no GR24 treatment. Similarly, RLD was 22.2% and 71.4% higher with GR24 treatment under moderate and severe drought stress, respectively, compared to plants without GR24 treatment.

**Table 1:** The effect of strigolactones on root biomass, total root length (TRL), root length density (RLD), root surface area (RSA) and root-shoot ratio (RSR) at different water treatments in dryland wheat

Water treatments	Chemical treatments	Root biomass (g plant^−1^)	TRL (m plant^−1^)	RLD (cm cm^−3^)	RSA (cm^2^ cm^−2^)	RSR
80% FC	CK	1.5^bc^	50.3^b^	1.9^b^	218.4^c^	0.11^a^
	+ GR24	1.7^cd^	51.4^b^	2.0^b^	223.8^c^	0.13^ab^
50% FC	CK	1.9^d^	57.6^c^	2.7^d^	231.5^d^	0. 15^bc^
	+ GR24	2.6^e^	63.8^d^	3.3^e^	249.2^e^	0.19^d^
30% FC	CK	1.1^a^	44.2^a^	1.4^a^	181.8^a^	0.14^b^
	+ GR24	1.7^bc^	49.2^bc^	2.4^c^	209.5^b^	0.17^cd^

Within a column, data presented are means ± standard deviation (*n* = 3). Different letters indicates significant differences for different water and chemical treatments (*p* < 0.05)

### Grain yield, yield components and water use efficiency

Drought stress significantly reduced the plant height and grain yield in dryland wheat. However, GR24 effectively mitigated the effects of moderate and severe drought stress on plant height, resulting in significantly taller plants compared to those without GR24 treatment (Table 2). Similarly, dryland wheat treated with GR24 showed a 34.9% increase in grain yield under moderate drought stress compared to plants without GR24 treatment. However, there was no significant difference in grain yield between plant treated with GR24 and those without under severe drought stress. Harvest index was not significantly affected by either water shortage or GR24 treatment. GR24 treatment led to a 21.5% and 33.3% increase in the water use efficiency of dryland wheat under moderate and severe drought stress, respectively. However, it had no significant effect on the water use efficiency of dryland wheat under well-watered conditions. Regardless of GR24 treatment, grain number per ear significantly decreased under both moderate and severe drought stress. Compared to untreated plants, GR24-treated wheat showed a significant increase in grain number under moderate drought stress, but no significant effect was observed under severe drought stress. There was no significant difference in the 1000-kernel weight between plants with and without GR24 treatment. Similar to grain number, the grain yield of dryland wheat was 34.9% higher under GR24 treatment than without GR24 treatment at the moderate drought stress. However, there was no significant difference in grain yield between plants treated with GR24 and those without under severe drought stress (Table 2).

**Table 2:** The effect of strigolactones on plant height, grain number, 1000-grain weight (TKW), grain yield, harvest index (HI), and water use efficiency for grain yield (WUE_G_) at different water treatments in dryland wheat

Water treatments	Chemical Treatments	Plant height (cm plant^−1^)	Grain number (plant ^−1^)	TKW (g)	Grain yield (g plant^−1^)	HI	WUE_G_ (g kg^−1^)
80% FC	CK	116.2^d^	167^d^	31.2^c^	3.82^d^	0.36^a^	1.07^a^
	+ GR24	110.3^cd^	159^d^	30.9^c^	3.61^d^	0.35^a^	1.17^a^
50% FC	CK	95.3^b^	103^b^	26.7^b^	2.32^b^	0.34^a^	1.21^ab^
	+ GR24	101.8^bc^	149^c^	28.7^bc^	3.13^c^	0.37^a^	1.47^c^
30% FC	CK	79.8^a^	76^a^	22.5^a^	1.84^a^	0.36^a^	1.02^a^
	+ GR24	85.6^ab^	82^a^	20.4^a^	1.91^a^	0.34^a^	1.36^bc^

Within a column, data presented are means ± standard deviation (*n* = 3). Different letters denote significant differences for different water and chemical treatments (*p* < 0.05)

## Discussion

### Strigolactones widened soil moisture threshold range of non-hydraulic root signaling and enhanced drought tolerance

This study showed that SLs significantly increased ABA concentration under drought stress (Fig. 1), indicating that SLs induced ABA accumulation. There was complex crosstalk between SLs and ABA during a drought stress environment (Liu et al. 2015; Min et al. 2019). A positive correlation between SLs and ABA has been observed in many plants under stress conditions (Aroca et al. 2013; Ruiz-Lozano et al. 2015; Min et al. 2019; Liu et al. 2020). The promotion of ABA synthesis by SLs is attributed to the up-regulation of NCED1, a key enzyme involved in ABA biosynthesis (Zhang et al. 2018; Min et al. 2019; Liu et al. 2020). The increase in ABA concentration was significantly related to the decrease of leaf *gs* under drought stress, which was involved in drought tolerance improvement, as confirmed in wheat (Du et al. 2012). Our study clearly showed that leaf *gs* was decreased after SLs application when SWC was at higher levels (73.4% FC in SLs application, 68.5% FC in CK) (Figs. 2 and 3). This result further confirmed that the application of SLs triggered an earlier closure of leaf stomata. This tendency broadened the threshold range of soil water content during the continuous operation of nHRS, compared to CK. SLs could not only regulate stomatal behavior in plants, but also improve plant drought resistance ability (Visentin et al. 2020). Although the application of SLs had not delayed the soil moisture threshold of leaf RWC, the soil water threshold range of nHRS was broadened due to the application of SLs in our study (Fig. 3). nHRS enables plants to sense soil drying and then respond to it and is often considered to be essential in regulating shoot growth, grain yield and water use efficiency (Davies and Zhang 1991; Xiong et al. 2006b; Batool et al. 2019). nHRS is recognized as a positive early warning response to drought stress in higher plants (Batool et al. 2019; Lv et al. 2019). Dryland wheat is grown in erratic soil water environments, and its yield formation is mostly regulated by nHRS under soil drying (Batool et al. 2019). Previous research showed that threshold range of nHRS was associated with increased drought resistance (Xiong et al. 2006a; Du et al. 2012; Lv et al. 2019). Wheat cultivars with wider threshold range of nHRS had the longest survival duration and better yield stability under moderate drought stress (Xiong et al. 2006a; Lv et al. 2019). Modern wheat cultivars had a wider threshold range of nHRS than old wheat varieties, which effectively improved crop yield and leaf dehydration tolerance (Du et al. 2012; Batool et al. 2019). SLs treatment reduced the lethal water potential and increased grain yield under moderate drought stress. Water use efficiency under both moderate and severe drought stress was also increased according to the results of this study. Our results might open a window for improving drought resistance, grain yield and water use efficiency of wheat by application of SLs to regulate early-warning response to drought stress.

### Strigolactones increased antioxidant enzyme activities and reduced the reactive oxygen species (ROS) and lipid membrane peroxide levels

Drought can reduce the assimilation of CO_2_, increase the electron transfer of photosynthetic electron carrier to O_2_, and increase reactive oxygen species (ROS), namely superoxide anion radical (O_2_^−^), hydrogen peroxide (H_2_O_2_) and hydroxyl radical (·OH) (Min et al. 2019). ROS, especially H_2_O_2_, acts as a signal in guard cells (Yin et al. 2022). The present study showed that ABA content was significantly increased when the nHRS was present, while ROS was still at a very low level and membrane lipid oxidation damage had not appeared (low level of MDA) under mild water stress (Fig. 5). These results suggested that stomatal closure is dependent on nHRS, which was the only early warning signal of imminent drought. When soil water content was at moderate stress level, the ROS production of dryland wheat leaves increased significantly with the intensification of drought stress (Fig. 5). Redox signals and ROS were the second messengers induced by nHRS (Fan et al. 2009; Batool et al. 2019). In the present study, the levels of ROS under SLs treatment were significantly lower than that without SLs treatment, while the activity of antioxidant enzymes was significantly higher than that without SLs treatment. These results might be related to the up-regulation of antioxidant-enzyme genes in plants mediated by SLs (Xu et al. 2023). These might also be attributed to the early priming the pre-existing defence system (Du et al. 2012). nHRS-mediated signaling increased ABA production and triggered ROS generation, thereby, led to up-regulation of antioxidant defense system (Batool et al. 2019). Plants balanced redox signaling in cells by regulating antioxidant enzymes of SOD, CAT, AXP and GR, which in turn improve their tolerance to drought stress (Upadhyaya et al. 2013; Batool et al. 2019). In our study, the higher activities of the antioxidant enzymes SOD, CAT, APX and GR were displayed in the SLs treatment (Fig. 6). SOD catalyzes the dismutation of O_2_^−^ to H_2_O_2_, whereas CAT, APX and GR are responsible for the removal of H_2_O_2_ (Li et al. 2018; Dong et al. 2022). Low O_2_^−^ and H_2_O_2_ production levels explained the low lipid damage in the GR24 treatment. This strong antioxidant system may have helped SLs treated plants reduce oxidative damage, thereby improving tolerance to moderate water stress (Jaleel et al. 2009; Du et al. 2012). This might also be an important reason for the higher yield of GR24 treated wheat under moderate drought stress. When the soil water content continued to decline to severe drought stress, plants transitioned from temporary wilting to permanent wilting, and the synthesis of antioxidant enzymes could not deal with the collapse of antioxidant defense system, and the enzyme activity started to decrease, which led to the excessive ROS production and intensification of membrane lipid peroxidation (Fan et al. 2009; Du et al. 2012). The application of SLs effectively reduced the content of MDA and reduced the damage of membrane lipid in dryland wheat caused by oxidative stress under severe drought stress. Likewise, Ma also previously showed that the application of SLs can reduce MDA content and reduce damage of membrane lipid (Ma et al. 2017). Although our study found that there was no significant difference in grain yield under severe drought stress between the treatments with SLs and without SLs, the application of SLs improved WUE and leaf desiccation tolerance (lower lethal leaf water potential) in dryland wheat under severe drought stress. This could serve as the evidence of improved drought-sensing signal of dryland wheat by SLs.

### Strigolactones optimized root system architecture

The root system is the major organ for plants to acquire water and nutrients and is associated with changes in yield and water use efficiency, especially drought environment (Fang et al. 2017). Root morphological traits are closely related to the yield formation and water use efficiency (Fang et al. 2017; Li et al. b). The role of SLs in shaping the root system architecture has been demonstrated in various species, including Arabidopsis, pea, grass, rice, and tomato, with most of studies done on Arabidopsis (Sun et al. 2022; Al-Amri et al. 2023). In our study, the application of SLs significantly increased root biomass, root length density and root to shoot ratio in dryland wheat under moderate and severe drought stress. The effects of SLs were mainly observed on root length, root length density, adventitious roots and root hair formation (Matthys et al. 2016). The increase in root biomass may be attributed to the regulation of root length and adventitious root development by SLs according to the data from this study. Increasing root biomass is an important drought-resistant strategy under drought conditions (Li et al. 2022a). Higher root biomass in the SLs treatment was conducive to the absorption of more water and nutrients, which led to an increase in yield and water use efficiency. Root length density and root to shoot ratio have been found to be associated with drought adaptation and increased productivity under drought stress (Chen et al. 2021; Li et al. b). Dryland wheat with higher root length density under SLs treatment was thought to increase soil exploration and an effective utilization of soil moisture through enhanced assimilation investment in root length (Wang et al. 2022). Under moderate drought stress, the higher grain yield in the SLs treatment was likely related to increased soil exploration and an effective utilization of soil moisture. The application of SLs may affect root hair development and root anatomy of dryland wheat under drought stress, thus affecting yield and water use efficiency. Further studies on the effects of SLs on root hair length, density and root anatomy are needed in subsequent experiments.

### Breeding perspectives and study limitations

Drought stress is one of the most important environmental factors affecting plant growth and development, and limiting plant production. Plants can respond and adapt to drought stress by using pre-existing and induced plant defenses to mediate their adaptation to stress conditions, thereby improving yield and WUE (Du et al. 2012). In recent years, many new techniques, including conventional breeding and molecular breeding, have been used to improve crop drought resistance and yield (Wang et al. 2021). Jakab et al. (2005) and Du et al. (2012) proposed that triggering earlier warning of drought in crops and activating antioxidant defense systems are also new and very effective ways to combat drought, compared to the manipulation of the genome. In our study, the application of SLs triggered earlier drought-sensing, and effectively primed pre-existing defense pathways, thus improving grain yield under moderate drought stress and WUE under moderate and severe drought stress in dryland wheat. Therefore, SLs induced drought resistance through early-warning response to drought and priming the activation of the antioxidant defense mechanism have been proposed as a way of generating crop varieties with an enhanced defensive capacity against drought stress. The SLs treatment only increased grain yield under moderate drought stress but not under severe drought stress in this study. In addition to leaf spraying, there are other methods of application such as soil drenching, stem injection and seed priming that may improve the grain yield of dryland wheat under severe drought stress (Sedaghat et al. 2021). Future work should determine which method or combination of application methods and intervals between treatments is the most effective and cost effective in increasing yields under water limited conditions. 

## Conclusion

SLs application triggered an earlier stomatal closure in the wheat plant and increased the activity of antioxidant defense enzymes, resulting in a wider threshold range of SWC and higher grain yield and water use efficiency. This study clarified the effect of strigolactones on the non-hydraulic root signals and antioxidant defense, which is beneficial for wheat to pre-sense drought and improve the defense ability of wheat plants against drought stress, thus enhancing grain yield and water use efficiency. This study provided an important theoretical basis for improving the early warning of drought stress, optimize the antioxidant defense system and enhance grain yield and water use efficiency of dryland wheat through exogenous plant hormones.

## Material and methods

### Plant materials and growth conditions

The wheat variety tested in this study was "Changhan 58", a semi-winter wheat cultivar mainly planted in Yangling, Shaanxi Province of China, with an average annual yield of 5500 kg·ha^−1^. "Changhan 58" was a variety derived from a high-generation cross between "PH82-2" as the female parent and an introducing material, "Changwu 112", as the male parent. The experiment was conducted at the institute of Soil and Water Conservation of Northwest A&F University, Yangling, Shaanxi Province of China (34°16′56.24″N, 108°04′27.95″E; 460 m asl). The test site represents the semi-arid and semi-humid climate region in northwestern China, with a total annual precipitation of 587 mm and potential evaporation of 884 mm. The variety was grown under a rainout shelter (20 m long × 5 m wide × 5.7 m high) that was closed during rain events. Plastic pots (280 mm diameter × 300 mm high) containing 10 kg of air-dried, homogenized (2 mm sieve) soil and potting compost (soil: potting compost = 1: 1, v/v) were used for the test. The soil was taken from a nearby loess-textured field. According to soil tests, 2.35 g urea (N), 1.81 g calcium superphosphate (P), 0.67 g potassium sulfate (K) were applied per pot to ensure adequate nutrition before sowing (Du et al. 2012). The soil mixture had a field capacity (FC) of 43.5%. Seeds were germinated in an incubator for 24 h, and 15 vernalized seeds were sown per pot. After emergence, seedlings were thinned to 10 plants per pot, and plants were irrigated daily to maintain the soil at 85% of FC.

At 30 d after sowing (DAS), the pots were transferred from the rainout shelter to a growth chamber. The growth chamber was set at 22/15℃ (day/night) temperature, 50 ± 5% relative humidity, and 300 µmol m^–2^ s^–1^ photosynthetically active radiation.

### SLs treatment

At 30 d after sowing (DAS), 15 mL of 10 μM synthetic analogues of SLs (GR24) was applied as a foliar spray to the leaves of all plants in each pot, three times (morning, noon and evening; until the leaves were dripping wet) daily for 3 d. The amount of GR24 applied was determined based on the results of the preliminary trail (Exogenous 10 μM GR24 could significantly improve wheat grain yield and water use efficiency). CK plants were sprayed with the same amount of distilled water. All plants were well watered during the 3-day foliar treatments. The commencement of SLs treatment is considered to be day 0.

### Experiment 1. Progressive soil drying

The effect of SLs on the physiological and biochemical responses of wheat was investigated by applying two watering treatments from 30 DAS: (1) water was withheld after the SLs treatment until the permanent wilting point, and (2) pots maintained at 80% FC (CK). The treatment was randomly implemented across six harvest times, with each treatment replicated in three blocks, resulting in 72 pots for each treatment. The replicate blocks were repositioned in the growth cabinet every 3d.

### Stomatal conductance and relative water content

Given that the soil dried after water was withheld, all pots were weighed daily to the target soil water content. Soil water content (SWC) is expressed as a percentage of available water between FC and dry soil. Leaf stomatal conductance (*gs*) and leaf relative water content (RWC) were measured on one mature, non-senescent leaf in each of the three replicate pots between 8:00 to 10:30 am, using the Li-6400 portable photosynthesis system (Li-Cor, Lincoln, NE, USA). The stomatal conductance was an average of four readings per leaf. The RWC was then measured at a similar position on the leaf used for *gs* measurement according to the method proposed by Turner (1996). The leaves were cut and weighed immediately to determine the fresh weight (FW). The leaves were floated on distilled water for 8 h in a petri dish in the dark at 25℃, surface dried with a paper towel, and weighed to determine the turgid weight (TW). The dry weight (DW) was measured after the leaves were oven-dried at 80℃ for 24 h. RWC was calculated according to the following equation:

$$\mathrm{RWC}\;=\;\lbrack(\mathrm{FW}-\mathrm{DW})\;/\;(\mathrm{TW}-\mathrm{DW})\rbrack\;\times\;100\%$$

A sequence of water gradient values was designed to measure the non-hydraulic root signals (nHRS) and hydraulic root signals (HRS), in which the SWC was defined at levels 30, 35, 40, 45, 50, 55, 60, 65, 70, 75, and 80% FC, with a fluctuation of 2.5% at each level. Leaf RWC and *gs* were monitored for 3 d prior to the start of soil drying to ensure that equilibrium with chamber conditions was established (Du et al. 2012). The collected data of plants were used to infer relations between leaf RWC or *gs* and SWC. The nHRS was judged to begin when there was a significant reduction in leaf *gs* without change in leaf RWC, and ended when both *gs* and RWC decreased simultaneously (the onset of hydraulic signals, HRS). This criterion was used to determine the SWC at which the nHRS started to appear and disappear, compared with the 80% FC group. Permanent wilting point (PW) was recorded when the leaves failed to recover overnight (Du et al. 2012).

### Abscisic acid (ABA) extraction and quantification

Given that the soil dried after withholding water, biochemical measurements of leaf tissue samples were collected in six times as follows: (1) SWC was at 80% FC (before the start of the soil drying); (2) SWC was decreased to 70% FC; (3) SWC was decreased to 60% FC); (4) SWC was decreased to 50% FC; (5) SWC was decreased to 40% FC); (6) SWC was decreased to 30% FC.

The extraction and purification of ABA were referred to the methods of Du et al. (2012) and Bollmark et al. (1988). Leaf samples were ground and extracted with ice cold 80% methanol (v/v) containing 1 mM butylated hydroxytoluence to avoid oxidation, and then stored overnight at 4°C. The extracts were then centrifuged at 10,000 g for 900 s at 4°C. Mixed supernatants were passed through Chromosep C_18_ columns (C_18_ Sep-Park Cartridge, Waters, Millford, MA, USA), and prewashed with 10 mL of 100% and 5 mL of 80% methanol, respectively. The exudate was collected and dried by evaporation with nitrogen. The residues were dissolved in 1.6 mL of phosphate-buffered saline (PBS) containing 0.1% (v/v) Tween 20 and 0.1% (w/v) gelatin (pH 7.5) for analysis by enzyme linked immunosorbent assay (ELISA), based the method of Yang et al. (2001) and Du et al. (2012).

### Lipid peroxidation and reactive oxygen species

The level of lipid peroxidation was estimated by the content of malondialdehyde (MDA), a product of lipid peroxidation (Du et al. 2012). Leaf sample (1 g) was homogenized in 10 mL of 10% trichloro acetic acid (TCA). The homogenate was centrifuged at 1800 g for 10 min. To a 2.0 mL aliquot of the supernatant, 2.0 mL of 0.6% thiobarbituric acid in 10% TCA, was added. The mixture was heated at 100°C for 15 min and then quickly cooled in an ice bath. After centrifugation at 4000 g for 10 min, the absorbance of the supernatant was recorded at 532, 600 and 450 nm for the estimation of nmol MDA g^−1^ dry weight.

The production of O_2_^−^ was measured by monitoring the nitrite formed from hydroxylamine in the presence of O_2_^−^ (Elstner and Heupel 1976). Leaf segments (0.5 g) were homogenized with 5 mL of 50 mM potassium phosphate (pH 7.8) and centrifuged at 5000 g for 10 min at 4°C. The mixture containing 1 mL of 1 mM hydroxylamine hydrochloride and 1 mL of supernatant was incubated at 25°C for 1200 s, before adding 17 mM sulphanilamide and 7 mM α-naphthylamine, and reacted at 25°C for 20 min. The absorbance of the aqueous solution was measured for the production of O_2_^−^ at 530 nm. H_2_O_2_ was measured as the absorbance of the titanium peroxide complex at 415 nm according to the method of Du et al. (2012).

### Enzyme assays

Superoxide dismutase (SOD) activity was determined by estimating the ability of nitro blue tetrazolium to inhibit the photochemical reduction. The absorbance was read at 560 nm (Du et al. 2012). Catalase (CAT) activity was monitored at 240 nm for 180 s according to Aebi (1984). Ascorbate peroxidase (APX) activity was measured by monitoring the changes in absorbance at 290 nm (with an extinction coefficient of 26.6 mM^−1^ cm^−1^). The enzyme extract (200 mL) was mixed with 50 mM potassium phosphate buffer (pH 7.0), ascorbic acid (0.5 mM) and H_2_O_2_ (0.1 mM) to yield a final volume of 1 mL (Amako et al. 1994). Glutathione reductase (GR) activity was assayed following the method described by Schaedle and Bassham (1977) by tracking NADPH oxidation at 340 nm for 60 s.

### Lethal leaf water potential

Lethal leaf water potential was measured following Augé et al. (1998) for plants whose soil was dried for more than 20 d determined between 8:00 am and 10:00 am, using a pressure chamber (PMS Instrument Company, Albany, OR, USA). The value of lethal leaf water potential was the data when leaves failed to recover the night following their measurement and died.

### Experiment 2: Long term water stress

Long term water stress was conducted to assess the effect of SLs treatment on yield and yield components of wheat plants exposed to three levels of water regimes. The wheat plants were watered by weight to maintain SWC at 80% FC from sowing to seedling stage (30 d before sowing). Thereafter, three levels of water availability were imposed: (1) One third pots were maintained at 80% FC from 30 d to maturity; (2) One third pots were maintained at 50% FC from 30 d to maturity; (3) One third pots were maintained at 30% FC from 30 d to maturity. At 40 d (seedling stage) and 120 d (jointing stage) after sowing, 15 mL of 10 μM GR24 per pot was applied to the leaves as a foliar spray three times a day for 3 d as the same in Experiment 1.

### Yield and water use efficiency

At maturity, the plant height was measured from the pot surface to the top of the spike (excluding the awns). The total tillers and productive tillers (tillers with at least one filled seed) were determined for each plant. The aboveground parts were harvested, oven-dried at 80℃ to a constant weight and weighed. The spikes were threshed, and the grains were re-dried at 50℃ to determine yield. The following variables were calculated: harvest index (HI) = grain yield / aboveground biomass; water use efficiency for grain (WUE_G_) = grain yield / water use from sowing to maturity. Water use was calculated from the water added to each pot from sowing to harvest.

### Root morphological traits

At maturity, each pot after cutting the shoots at the crown level was filled with water to loosen the soil. A round-shaped shaker was used to gently vibrate the mixture until the roots were separated from the soil particles. The roots were washed carefully to avoid losing any fine roots. Clean roots were then scanned at 600 pixels per mm and root images were analyzed using WinRHIZO (Regent Instruments Inc., Québec City, QC, Canada) to determine total root length (TRL) and root surface area (RSA) as described by Himmelbauer et al. (2004). Root samples were then oven dried at 80℃ to a constant weight and weighed after being scanned.

### Statistical analysis

Two ways ANOVA (Water treatments and SLs treatments) was used to analyze data from Experiments 1 and 2. When ANOVA showed significant effects, treatment mean differences were compared by the Least Significant Difference (LSD) values at *p* = 0.05. All data analyses were conducted using the Statistical Analysis System (SAS Institute. 2018). The changes of *gs* and leaf RWC as a percentage of the controls (Fig. 2) was fitted as follows:

$$\mathrm{Y}=100/\{\left[1+\mathrm{exp}\left(-x-{x}_{0}\right)\right]/b\}$$

where ${x}_{0}$ is *gs* and leaf RWC under the condition of sufficient water supply; $x$ is *gs* and leaf RWC after water was withheld in dryland wheat.

The relative values of *gs* and leaf RWC (Fig. 3) was calculated as follows:

$$1\mathrm{\;if\;Ci}\le \mathrm{SWC}\le\;1$$

$$1-\mathrm{A}\times \left(\mathrm{SWC}-\mathrm{Ci}\right)\mathrm{\;if\;SWC}\le \mathrm{Ci}$$

where A is the slope of the linear equation and Ci is the threshold of SWC at which the measured traits started to diverge. Model of ‘linear-plateau’ and ‘broken stick’ regression were sued to assess A and Ci (PROCNLIN of PC SAS). The coefficient of determination (r^2^) was calculated for each curve as 1–SSE/CSS, where SSE is the residual sum of squares and CSS is the corrected total sum of squares (Liu et al. 2003; Tom and Lesperance 2003).

## Data Availability

The data and material that support the findings of this study are available from the corresponding author upon reasonable request.

## Abbreviations

nHRS: Non-hydraulic root signal
HRS: Hydraulic root signal
